# Are high-level aftereffects perceptual?

**DOI:** 10.3389/fpsyg.2015.00157

**Published:** 2015-02-19

**Authors:** Katherine R. Storrs

**Affiliations:** Perception Lab, School of Psychology, University of QueenslandBrisbane, QLD, Australia

**Keywords:** psychophysical methods, aftereffects, anchoring effect, response bias, contrast effect, neural adaptation, adaptation, psychological

## A high-level aftereffect, and two ways to explain it

Imagine an experiment in which you show someone pictures of a computer-generated face displaying random expressions, ranging from happy, through neutral, to sad. You tell the participant that she must classify each picture as being either “happy” or “sad,” and you note the point on the expression continuum at which she switches from mostly-“happy” classifications to mostly-“sad.” Then, you ask her to repeat the task, but between each picture you have her feel, with her hands but out of sight, the contours of a smiling face mask. You find that her category boundary has shifted: she now classifies more of the pictures as “sad.” This result (reported by Matsumiya, 2013) is an example of an aftereffect, in which adaptation to one input (the mask) has altered responses to subsequent inputs (the images). It is “high-level” in the sense that the adapting and test stimuli have little overlap in their initial sensory encoding (they are presented in separate modalities). There are at least two ways to interpret this finding.

### A decisional bias?

The presence of the smiling mask may have altered the participant's strategies or criteria for labeling the expression images. For instance, she may now be consciously or unconsciously using the rule: “if in doubt, say the expression was different from that of the mask.” This interpretation places the effect within the extensive catalog of contrast effects in the cognitive and social psychological literature. For example, people judge moderately qualified job applicants as being less qualified after reading the résumé of a highly qualified competitor (Hakel et al., 1970; Wexley et al., 1972).

### A perceptual bias?

Alternatively, feeling the smiling mask may have changed how the test pictures *look* to the participant. Although she uses the same strategies and criteria to arrive at her decision, the boundary between “happy” and “sad” expressions falls at a different point because her encoding of the stimuli has changed. According to this interpretation, Matsumiya's (2013) effect is an example of a visual aftereffect, akin to the temporary illusions induced by prolonged exposure to a particular color (Webster, 1996), motion direction (Addams, 1834; Anstis et al., 1998), orientation (Gibson and Radner, 1937), or spatial frequency (Blakemore and Sutton, 1969). Such aftereffects can be visually striking, and have been linked to changes in the responsiveness of neurons selective for the properties of the inducing stimulus (neural adaptation—see, e.g., Kohn, 2007; Webster, 2012).

Matsumiya's (2013) effect joins a growing body of aftereffects between increasingly abstractly-related adapting and test stimuli. For example, people are more likely to report an androgynous face as being male after viewing a female face (or *vice versa*; Webster et al., 2004), or even after viewing female bodies (Ghuman et al., 2010) or stereotypically female objects (Javadi and Wee, 2012). Analogous effects occur between facial images depicting different identities (Leopold et al., 2001), races, expressions (Webster et al., 2004), ages (Schweinberger et al., 2010), and geometric distortions (Webster and Maclin, 1999). Further examples of high-level aftereffects abound outside of face perception: after receiving downwards-moving tactile stimulation to their hands, people more often judge an oscillating visual grating to be drifting upwards (Konkle et al., 2009); after looking at a looming visual pattern, people more often judge a steady auditory tone to be receding in depth (Kitagawa and Ichihara, 2002); and after seeing a series of urban landscapes, people more often judge semi-rural landscapes to be “natural” (Greene and Oliva, 2010).

In each of these reports, the aftereffect is interpreted as a *perceptual* bias due to neural adaptation. If this interpretation is correct, high-level aftereffects may provide exciting tools to investigate how complex stimulus properties are encoded, just as “low-level” aftereffects have for simpler stimulus properties (Barlow and Hill, 1963; Blakemore and Campbell, 1969; Mollon, 1974; Thompson and Burr, 2009; Thompson and Burr, although see also Hegde, 2009 for a note of caution). Already, face aftereffects have been widely used to study the encoding of faces (e.g., Leopold et al., 2001; Rhodes and Jeffery, 2006; Susilo et al., 2010; Zhao et al., 2011; Storrs and Arnold, 2012; McKone et al., 2014). If high-level aftereffects are decisional biases, on the other hand, they may all have a similar origin within amodal cognitive processes and tell us little about the representation of any particular stimulus property. So how can one distinguish perceptual from decisional biases?

## Methods to distinguish perceptual from decisional biases

### The bias manifests in a “criterion free” task

With the exception of Webster and Maclin (1999), each of the high-level aftereffects above was demonstrated using a “method of single stimuli” (MSS). In an MSS task, a single test stimulus is shown on each trial and the observer classifies it as belonging to one of two categories. The placement of the category boundary is determined both by the participant's sensory evidence and by her criteria for applying each of the response labels to that evidence (see Green and Swets, 1966; Farell and Pelli, 1999; Kingdom and Prins, 2010). Changes in criteria can therefore produce exactly the same pattern of response shifts as changes in perception, making MSS data ambiguous (Green and Swets, 1966; Gescheider et al., 1970; Morgan et al., 2011, 2013; Yarrow et al., 2011). Why then have many papers in recent years claimed to report novel *perceptual* aftereffects on the basis only of MSS data?

Unfortunately, the best psychophysical methods to measure *perceptual* experience are unsuited to high-level aftereffects. Visual appearance can be measured without relying on semantic labels or remembered reference stimuli only if there exists an *unadapted (or differently adapted) location* in the visual field. Adaptation to simple properties, such as orientation, contrast, and spatial frequency, produces aftereffects localized to within a few degrees of the adaptor (Gibson, 1937; Williams et al., 1982; Ejima and Takahashi, 1985). A test stimulus can then be shown within the affected region while a reference stimulus is shown in an unaffected region. The point of subjective equality (PSE) between adapted and unadapted locations is quantified by having the observer adjust the test to match the reference, indicate whether or not the two appear the same, or decide which location contains the “stronger” signal along some dimension (see Kingdom and Prins, 2010). This last task is known as a two-alternative forced-choice (2AFC, or more generally, *n*AFC).

Isolating *perceptual* bias is still not straightforward. If one shows the same tilted grating in an adapted and unadapted location and asks “which is tilted further clockwise?” (a simple 2AFC), a strategy of picking the stimulus in the adapted location when unsure could produce a shift in PSE between baseline and adaptation trials (Schneider and Komlos, 2008; Morgan, 2013, 2014; Jogan and Stocker, 2014). Such problems can be alleviated by elaborations to the *n*AFC task, such as varying the reference stimulus from trial-to-trial so that a perceptual bias predicts *opposite* PSE shifts for different reference stimuli (Morgan, 2013, 2014; Morgan et al., 2013) and presenting two reference stimuli in unadapted locations, from which the participant selects the one most similar to a test shown in the adapted location (Jogan and Stocker, 2014).

While there is no objective way to measure a subjective perceptual bias, *n*AFC methods with multiple reference stimuli come closest to providing a measure uncontaminated by decisional criteria. Unfortunately they are only practical when the aftereffect is localized to the adapted location. The position-dependence of most high-level aftereffects is unknown, but it seems likely that some (e.g., cross-modal aftereffects) are spatially global.

### The bias is mediated by properties of early sensory neurons

The magnitude of a perceptual bias is often mediated by the receptive field properties of early visual neurons. Adaptation at one retinal location may not affect tests elsewhere (see above), or adaptation in one eye may not affect tests seen with the other (e.g., McCollough, 1965). The bias may even occur when the adapting stimulus is suppressed from awareness (e.g., Blake and Fox, 1974). These indicators are of limited use in the present case, though, as high-level adaptation may not be mediated by properties of early visual neurons.

### The bias is accompanied by objectively-measured sensitivity changes

“Low-level” visual aftereffects are often accompanied by reduced sensitivity to detect the adapted properties. For example, the image contrast required to detect a grating pattern is selectively raised for patterns with a similar orientation and spatial frequency to the adaptor (Blakemore and Campbell, 1969; see also Levinson and Sekuler, 1980; Krauskopf et al., 1982). Changes may also be found in discrimination sensitivity (e.g., Regan and Beverley, 1985; Clifford et al., 2001).

Selective changes in sensitivity near an adapted value constitute reasonable evidence for changes in sensory encoding—they often accompany low-level aftereffects, are predicted by models based on neural adaptation (e.g., Blakemore and Campbell, 1969; Clifford et al., 2001; Kohn, 2007), and not easily explained in terms of decisional bias. *n*AFC tasks can provide objective measures of sensitivity (Green and Swets, 1966; Farell and Pelli, 1999) even when there is no unadapted visual field location. In the domain of face aftereffects, there is some evidence for improved discrimination near an adapted face (Rhodes et al., 2010; Oruc and Barton, 2011), although other researchers have found no changes in sensitivity (Rhodes et al., 2007; Ng et al., 2008).

### The bias fails to manifest in an “El greco task”

Since the opthalmologist Beritens proposed that an astigmatism was to blame for the oddly elongated figures painted by the artist El Greco, many have pointed out the fallacy in his theory: any optical distortion El Greco experienced must have applied equally to both his subjects and his own paintings (Rock, 1966; Anstis, 2002; Firestone, 2013). Likewise, if an adaptation-induced bias is a literal change in how things look, it should apply equally to the test and to any reference against which the observer judges it (Firestone and Scholl, 2013). After adaptation, an observer could be shown a test stimulus, then asked to adjust or select a reference stimulus to match it. Any bias shown in this “El Greco task” is likely of a cognitive rather than perceptual origin.

## Questionable methods to distinguish perceptual from decisional biases

### The bias has similar temporal dynamics to a perceptual one

Several authors (Leopold et al., 2005; Ghuman et al., 2010; Matsumiya, 2013) show that the magnitudes of their respective aftereffects increase logarithmically with the duration of the adapting stimulus. This is similar to the temporal dynamics of tilt (Magnussen and Johnsen, 1986) and motion (Hershenson, 1989) aftereffects, and is presented as evidence that the high-level aftereffects in question share a common mechanism with low-level aftereffects. *A priori*, the fact that two pairs of variables are related to one another by similar functions is poor evidence that they are subserved by similar mechanisms (a sum of money accumulating compound interest also increases logarithmically with time). Temporal dynamics may turn out to have diagnostic value, but only if effects deemed perceptual on other grounds have reliably different temporal dynamics from those deemed decisional. These data do not yet exist.

### The bias manifests in some conditions but not others

Kitagawa and Ichihara (2002) find that although viewing a looming visual pattern causes participants to judge a steady auditory tone as receding in depth, hearing a tone increasing in volume has no effect on visual judgements. The authors argue that this selectivity for particular adaptor-test pairings indicates a perceptual origin (Van der Burg et al. (2013) present a similar argument). This relies on the assumption that all adaptor-test pairings should be equally effective in inducing shifts in decisional criteria—it is not obvious why this should be the case.

## Conclusion

Much of the interest in high-level aftereffects depends on claims that, like colored afterimages or motion aftereffects, they involve literal changes in how the world looks, feels or sounds. Such changes in sensory encoding may help us understand how the brain represents complex stimulus properties and integrates information across modalities. However, most high-level aftereffects have so far been demonstrated only as biases in how people classify stimuli during method-of-single-stimulus tasks, and are therefore equally consistent with changes in amodal decision-making processes.

### Conflict of interest statement

The author declares that the research was conducted in the absence of any commercial or financial relationships that could be construed as a potential conflict of interest.
